# TFRC–RNA interactions show the regulation of gene expression and alternative splicing associated with IgAN in human renal tubule mesangial cells

**DOI:** 10.3389/fgene.2023.1176118

**Published:** 2023-07-20

**Authors:** Jian-Si Li, Xiao Chen, Ailing Luo, Dong Chen

**Affiliations:** ^1^ Department of Nephrology, The Second Affiliated Hospital of Harbin Medical University, Harbin, China; ^2^ Heilongjiang Provincial Hospital Affiliated to Harbin Institute of Technology, Harbin, China; ^3^ Center for Genome Analysis, Wuhan Ruixing Biotechnology Co., Ltd., Wuhan, China

**Keywords:** IgAN, TFRC, RNA-binding protein, RNA-seq, iRIP-seq

## Abstract

**Introduction:** IgA nephropathy (IgAN) is the most common primary glomerular disease (PGD) which could progress to renal failure and is characterized by aberrant IgA immune complex deposition. Transferrin receptor1 (TFRC), an IgA receptor, is a potential RNA binding protein (RBP) which regulates expression of genes positively associated with the cell cycle and proliferation and is involved in IgAN. Molecular mechanisms by which TFRC affects IgAN development remain unclear.

**Methods:** In this study, TFRC was overexpressed in human renal tubular mesangial cells (HRMCs) and RNA-sequencing (RNA-seq) and improved RNA immunoprecipitation sequencing (iRIP-seq) were performed. The aim was to identify potential RNA targets of TFRC at transcriptional and alternative splicing (AS) levels.

**Results:** TFRC-regulated AS genes were enriched in mRNA splicing and DNA repair, consistent with global changes due to TFRC overexpression (TFRC-OE). Expression of TFRC-regulated genes potentially associated with IgAN, including *CENPH, FOXM1, KIFC1, TOP2A, FABP4, ID1, KIF20A, ATF3, H19, IRF7, and H1-2*, and with AS, *CYGB, MCM7 and HNRNPH1*, were investigated by RT-qPCR and iRIP-seq data analyzed to identify TFRC-bound RNA targets. *RCC1* and *RPPH1* were found to be TFRC-bound RNA targets involved in cell proliferation.

**Discussion:** In conclusion, molecular TFRC targets were identified in HRMCs and TFRC found to regulate gene transcription and AS. TFRC is considered to have potential as a clinical therapeutic target.

## 1 Introduction

The common chronic glomerular disease, IgA nephropathy (IgAN), progresses to end-stage renal disease (ESRD) within 20–30 years of diagnosis for 30%–40% of patients, necessitating renal replacement therapy, such as kidney transplantation or dialysis (Lai et al., 2016). IgAN is characterized by proliferation of mesangial cells and deposition of IgA-dominated immune complexes in the mesangium. The “Multi-Hits” theory explains the aspects of IgAN pathogenesis with successive events, such as mucosal immune abnormalities, chronic inflammation (Novak et al., 2011), and complement activation (Tortajada et al., 2019), but specific pathogenic mechanisms remain unclear.

Glomerular mesangial cells (GMCs) are located between glomerular capillary loops, adjacent to endothelial cells (Sakai and Kriz, 1987), and connected with the glomerular basement membrane (GBM), where they participate in glomerular filtration (Kriz et al., 1990). Activation of these innate immune cells in response to macromolecular substances, immune complexes, or hypoxia has been associated with IgAN development. Autocrine and paracrine actions of histamine, serotonin, cytokines, interleukin, and cell growth factors produced by proliferating GMCs (Abboud, 2012) disturb the balance between mesangial matrix secretion and degradation, causing matrix accumulation in the glomeruli, glomerulosclerosis, and tubular interstitial fibrosis (Nogueira et al., 2017). Therefore, GMC behavior is essential to the prevention and treatment of IgAN.

Transferrin receptor 1 (TFRC or TfR1) is an IgA1 receptor required for erythropoiesis and nervous system development (Levy et al., 1999), which has been implicated in IgAN pathogenesis (Moura et al., 2001). TFRC is a transmembrane glycoprotein which mediates the endocytosis of iron ions from circulating ferritin, enhancing absorption. TFRC expression is significantly enhanced in IgAN (Moura et al., 2001), being overexpressed and deposited in renal tissues of IgAN patients and co-located with IgA1. The binding of IgA1 to TFRC induces TFRC overexpression in GMCs (Abbad et al., 2020).

TFRC has also been identified by a global RNA interactome capture to be an RNA-binding protein (RBP) (Castello et al., 2012). RBPs bind nuclear and cytosolic RNAs (Gerstberger et al., 2014), forming ribonucleoprotein particles (RNPs) (Castello et al., 2012), and influencing mRNA synthesis and degradation (Gerstberger et al., 2014; Marchese et al., 2016; Hentze et al., 2018). Previous studies have demonstrated the influence of TFRC on gene expression and AS in HeLa cells (Huang et al., 2020; Huang et al., 2022).

During the present work, molecular targets of RBPs and mechanisms involved were investigated by RNA immunoprecipitation (RIP) (Gilbert and Svejstrup, 2006) to ascertain whether TFRC regulates GMC signaling pathways through RNA binding. TFRC was overexpressed in human renal mesangial cells (HRMCs), and RNA-seq and improved RIP sequencing (iRIP-seq) were performed.

## 2 Materials and methods

### 2.1 Cell culture and transfection

Lentiviral vectors for TFRC overexpression were purchased from GenePharma (GenePharma Co., Ltd., Suzhou, China; transcript information: NM_003234.4, lentiviral vector: LV5 (EF-1a/GFP/Puro/Amp). HRMC lines (4200, ScienCell, United States) were cultured in the HRMC complete medium (4200, ScienCell, United States) with 100 U/mL penicillin, 100 μg/mL streptomycin (SV30010, HyClone, United States), and 10% fetal bovine serum (FBS) (10091148, Gibco, China) at 37°C and 5% CO_2_. Cells were transfected with lentivirus at a multiplicity of infection (MOI) 35, treated with 0.3 µg/mL puromycin and stable cell lines harvested for Western blotting and RT-qPCR analyses.

### 2.2 Gene expression

cDNA synthesis was performed using the reverse transcription kit (R021-01, Vazyme, China) in a thermocycler (T100, Bio-Rad, United States) at 37°C for 15 min. qPCR was performed using ABI QuantStudio 5 with an initial denaturation step at 95°C for 10 min, followed by 40 cycles of denaturation for 15 s at 95°C, annealing for 1 min at 60°C, and extension for 1 min at 60°C. The transcript level was normalized to glyceraldehyde-3-phosphate dehydrogenase (GAPDH), and fold change was analyzed by the 2^−ΔΔCT^ method (Livak and Schmittgen, 2001). Student’s *t*-test was used to compare test and control samples of three independent replicates using GraphPad Prism software (version number8.0, San Diego, CA). (q)PCR primers are presented in Supplementary Table S1.

### 2.3 Western blotting

HRMCs were lysed in ice–cold RIPA buffer (PR20001, Proteintech, China) containing protease inhibitor cocktail (PIC) (4693116001, Sigma, United States) and incubated on ice for 30 min. Samples were boiled with protein loading buffer (P1040, Solarbio, China) for 10 min, loaded onto 10% SDS-PAGE gel, and transferred onto 0.45 mm PVDF membranes (ISEQ00010, Millipore, United States). Membranes were blocked at room temperature for 1 h and incubated at 4°C overnight with primary antibodies increased against FLAG tags (anti-FLAG, 1:2,000, antibodies generated in rabbits, F7425, Sigma, United States) and GAPDH (1:1,000, antibodies generated in rabbits, A19056, ABclonal) and with horseradish peroxidase-conjugated secondary antibodies (anti-rabbit, 1:5,000, SA00001-2, Proteintech, China) at room temperature for 45 min. Membranes were visualized by chemiluminescence using an enhanced ECL reagent (P0018FM, Beyotime, China).

### 2.4 RNA extraction and sequencing

Total RNA was extracted with TRIzol (15596-018, Ambion, United States) and purified twice with phenol–chloroform and RQ1 DNase treatment (M6101, Promega, Madison, WI, United States) to eliminate DNA. Absorbance at 260/280 nm (A260/A280) was read on a SmartSpec Plus instrument (Bio-Rad, United States) to quantify RNA and integrity assessed by 1.5% agarose gel electrophoresis.

Three biological replicates were prepared for TFRC-OE and NC samples. RNA-seq libraries were prepared from 1 μg total RNA using a VAHTS Stranded mRNA-seq Library Prep Kit (NR605-02, Vazyme, China), and polyadenylated mRNAs were purified, fragmented, and converted into double-stranded complementary DNAs (cDNAs). cDNAs were ligated to VAHTS RNA Adapters after end repair and A tailing, and products of 200–500 bps were produced during digestion with heat-labile uracil-DNA glycosylase (UDG). Single-stranded cDNAs were amplified, purified, quantified, and stored at −80°C. Then, 150 nt paired-end sequencing was performed using the Illumina NovaSeq 6000 system, following the manufacturer’s protocol.

### 2.5 RNA-seq raw data cleaning and alignment

Raw reads with ≥ 2-N bases were removed, and FASTX-Toolkit (Version 0.0.13) was used to remove adapters and low-quality bases. Short reads ≤16 nt were discarded. Clean reads were mapped to the GRCh38 genome by HISAT2 (Kim et al., 2015), allowing four mismatches. Approximately 98% of mapped reads had two or less mismatches. Finally, the unique matches were used to calculate gene read number and fragments per kilobase of transcript per million fragments mapped (FPKM) (Trapnell et al., 2010).

### 2.6 Differentially expressed genes

DEGs were screened out using the R Bioconductor package, DESeq2 (Love et al., 2014), and fold change (FC) > 2 or <0.5 and *p*-value <0.05 were set as cut-off values. Screening criteria were the enrichment of expression in target signaling pathways and previous literature reports of genes of interest, which were then shown to be enriched by the current data.

### 2.7 Alternative splicing

Alternative splicing events (ASEs) and regulated alternative splicing events (RASEs) were quantified by the ABLas pipeline (Jin et al., 2017; Xia et al., 2017). Nine types of ASEs were detected by ABLas based on splice junction reads, including exon skipping (ES), mutually exclusive 5′UTRs (5pMXE), mutually exclusive 3′UTRs (3pMXE), alternative 5′splice site (A5SS), alternative 3’splice site (A3SS), cassette exon, mutually exclusive exons (MXE), A3SS&ES, and A5SS&ES.

Alterations in the RBP-regulated ASE ratio were evaluated by Student’s *t*-test. A *p*-value cutoff corresponding to a false discovery rate (FDR) of 0.05 was considered to indicate significant differences in TFRC-regulated ASEs.

### 2.8 iRIP-seq preparation

HRMCs were UV irradiated at 400 mJ/cm^2^ (CL-3000, UVP, Germany) and lysed in ice–cold wash buffer (1× PBS, 0.1% SDS, 0.5% NP-40%, and 0.5% sodium deoxycholate) containing PIC (B14001, Bimake, China) and a 200-U/mL RNase inhibitor (2313U, TaKaRa, Japan), and incubated on ice for 30 min. The cell lysate was centrifuged at 10,000 rpm at 4°C for 10 min. Then, 1U/µL RQ I (M610A, Promega, Madison, WI, United States) was added to a final concentration of 0.05 U/µL with incubation at 37°C for 40 min. A stop solution was added to inhibit DNase, and cell debris was removed by centrifugation at 13,000 g at 4°C for 20 min. RNAs were digested by micrococcal nuclease (MNase) (2910A, Thermo Scientific, United States).

The immunoprecipitation supernatant was incubated overnight at 4°C with 5 μg of the FLAG antibody (80010-1-RR, Proteintech, China) and with A/G Dynabeads (26162, Thermo Scientific, China) for 2 h at 4°C. Beads were pulled down with a magnet and washed twice with lysis buffer, high-salt buffer (250 mM Tris 7.4, 750 mM NaCl, 10 mM EDTA, 0.1% SDS, 0.5% NP-40, and 0.5 deoxycholate), and PNK buffer (50 mM Tris, 20 mM EGTA, and 0.5% NP-40) before resuspension in elution buffer (50 nM Tris 8.0, 10 mM EDTA, and 1% SDS). Immunoprecipitated RBPs with crosslinked RNAs were isolated by heating at 70°C for 20 min, and 1.2 mg/mL of suspension was digested with proteinase K (B14001, Bimake, China) at 55°C for 120 min. RNAs were purified with TRIzol reagent (15596-018, Ambion, United States).

cDNA libraries were prepared with the KAPA RNA HyperPrep Kit (KK8541, Roche, Switzerland), following the manufacturer’s procedure, and 150 nt paired-end sequencing was performed by Illumina NovaSeq, according to the manufacturer’s instructions.

### 2.9 Data analysis

Sequences were aligned with the genome by HISAT2 (Kim et al., 2015); PCR duplicates were removed, and peaks, “called” by Piranha (Uren et al., 2012) and ABL in *in silico* random clustering (ABLIRC) to identify genomic GRCh38-binding regions (Xia et al., 2017). The process of peak calling was as follows: the whole genome was scanned with a 5-bp window and at 5 bp from the beginning of each chromosome. Peaks were identified by setting the depth of the first window at 2.5 times for eight consecutive windows on the genome or medium depth greater than 50. When eight consecutive windows were ≤4% of the maximum depth of this peak, the peak was brought to an end. Reads were randomly distributed 500 times, and the frequency of each peak’s depth counted to conduct significance analyses on identified peaks and screen the significant peaks with a *p*-value <0.05 or with a maximum depth of ≥10. Peak location abundance differences were analyzed with input samples as control. The peak with input abundance ≥4 times (adjustable parameter) was identified as the final combination peak to identify TFRC target genes. HOMER software was used to identify binding motifs of the IP proteins (Heinz et al., 2010).

### 2.10 Functional enrichment analysis

Gene Ontology (GO) terms and Kyoto Encyclopedia of Genes and Genomes (KEGG) pathway analyses were performed on the KOBAS 2.0 server to assign functional categories to the DEGs (Xie et al., 2011). Enrichment for each term was defined by hypergeometric tests and the Benjamini–Hochberg FDR procedure.

## 3 Results

### 3.1 TFRC-regulated HRMC gene expression

**FIGURE 1:**
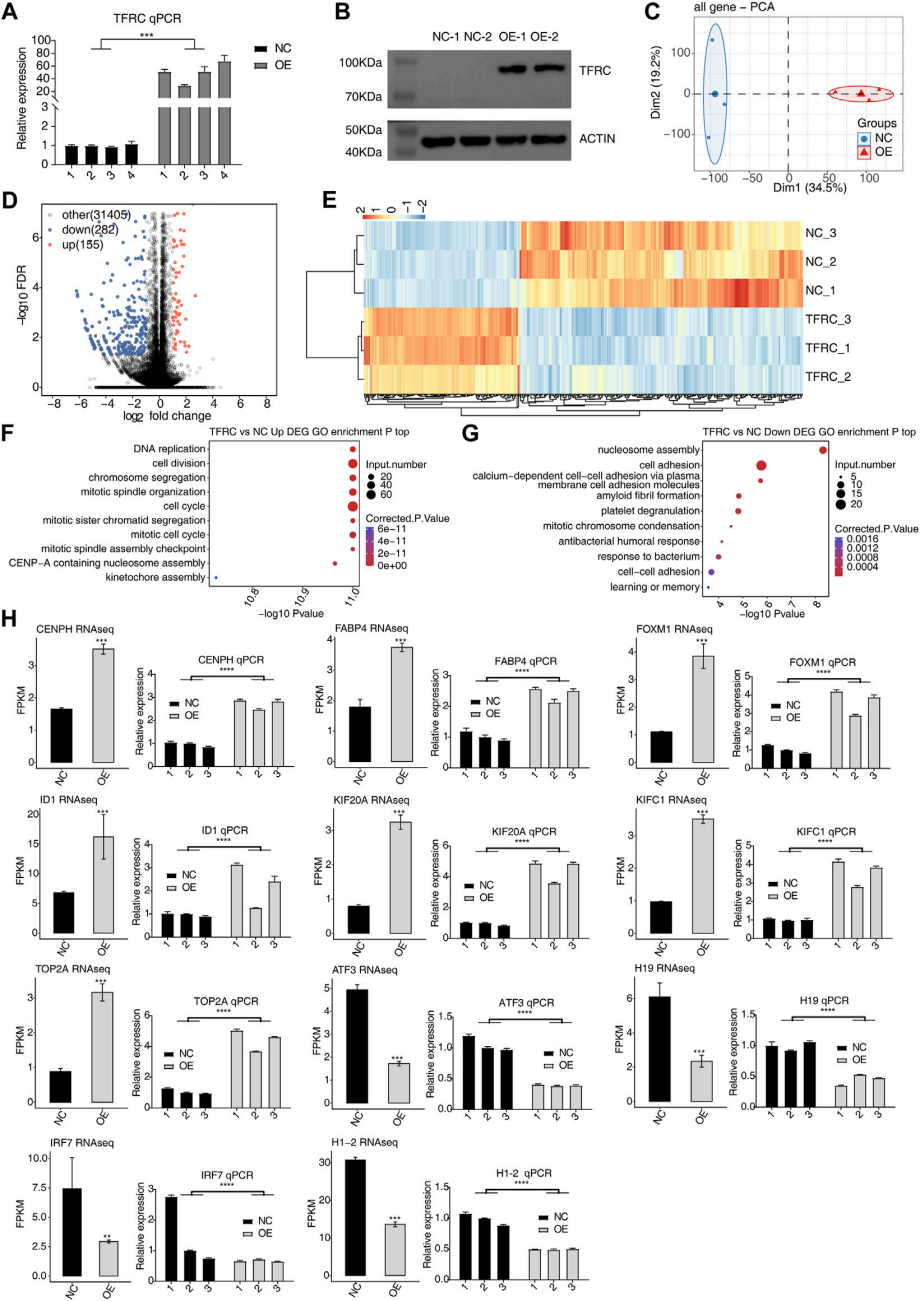
TFRC regulates the gene expression pattern in HRMCs. **(A)** RT-qPCR and **(B)** Western blotting increased TFRC expression by showing three and two biological replicates, respectively. Error bars represent SEM. **(C)** Principal component analysis showing the clear separation of the two sample groups based on normalized gene expression levels. **(D)** Volcano plot showing the TFRC-upregulated (red) and downregulated (blue) genes. Black points represent non-DEGs. **(E)** Hierarchical clustering heatmap showing the expression pattern of up- and downregulated DEGs. FPKM values of each gene were log2-transformed and normalized. **(F)** Bubble plot showing the top 10 GO BP pathways of upregulated DEGs. **(G)** Bubble plot showing the top 10 GO BP pathways of downregulated DEGs. **(H)** RNA-seq (left panel) and RT-qPCR (right panel) results for the 11 selected DEGs. Error bars represent SEM. *** *p*-value <0.001; ** *p*-value <0.01; Student’s *t*-test.

TfR1, encoded by TFRC, is essential to maintain the iron homeostasis in cells, thus playing critical roles in multiple biological processes (BPs) and diseases (Shen et al., 2018). Studies have proven TfR1’s transcriptional and post-transcriptional functions in HeLa cells (Huang et al., 2020; Huang et al., 2022). In this study, we overexpressed TFRC in HRMCs and identified its regulated transcriptome and RNA interactome to investigate the underlying molecular functions. After the TFRC overexpression plasmid was transfected into HRMCs, both Western blot and RT-qPCR experiments indicated that the overexpression samples of TFRC showed a higher level than those of the negative control (NC) samples (Figures 1A, B). Subsequently, RNA-seq was performed to identify the molecular targets of TfR1. Principal component analysis of all detected genes demonstrated the clear distinct expression patterns between TFRC-OE and NC samples (Figure 1C), indicating that TFRC-OE had a profound impact on the transcriptome profiles. DEGs were identified to explore the RNA targets and TFRC functions. TFRC-OE generated 155 up- and 282 down-regulated DEGs, with a marked tendency to downregulated DEGs (Figure 1D), suggesting that TFRC-OE could inhibit the gene transcriptional levels in HRMCs. The clustering heatmap demonstrated the consistent expression pattern within the three biological replicates and a clear distance between TFRC-OE and NC samples (Figure 1E). Functional enrichment analysis of these up- and downregulated DEGs also revealed their distinct enriched BPs. Upregulated DEGs were generally enriched in the cell cycle and proliferation-associated pathways, including DNA replication, cell division, and cell cycle-related pathways (Figure 1F), while downregulated DEGs were mainly enriched in the chromosome structure and cell adhesion-related pathways (Figure 1G). Both up- and downregulated DEGs were enriched in chromosome structure pathways, suggesting that TFRC-OE had a significant influence on the cell cycle and proliferation in HRMCs. Eventually, several DEGs were selected to show the differences between NC and TFRC-OE samples, including four downregulated and seven upregulated DEGs. To validate the dysregulated expression levels of these DEGs, the RT-qPCR experiment was performed. The RNA-seq and RT-qPCR results were highly consistent. All these genes selected were successfully validated (Figure 1H).

### 3.2 TFRC-regulated AS of HRMC genes

**FIGURE 2:**
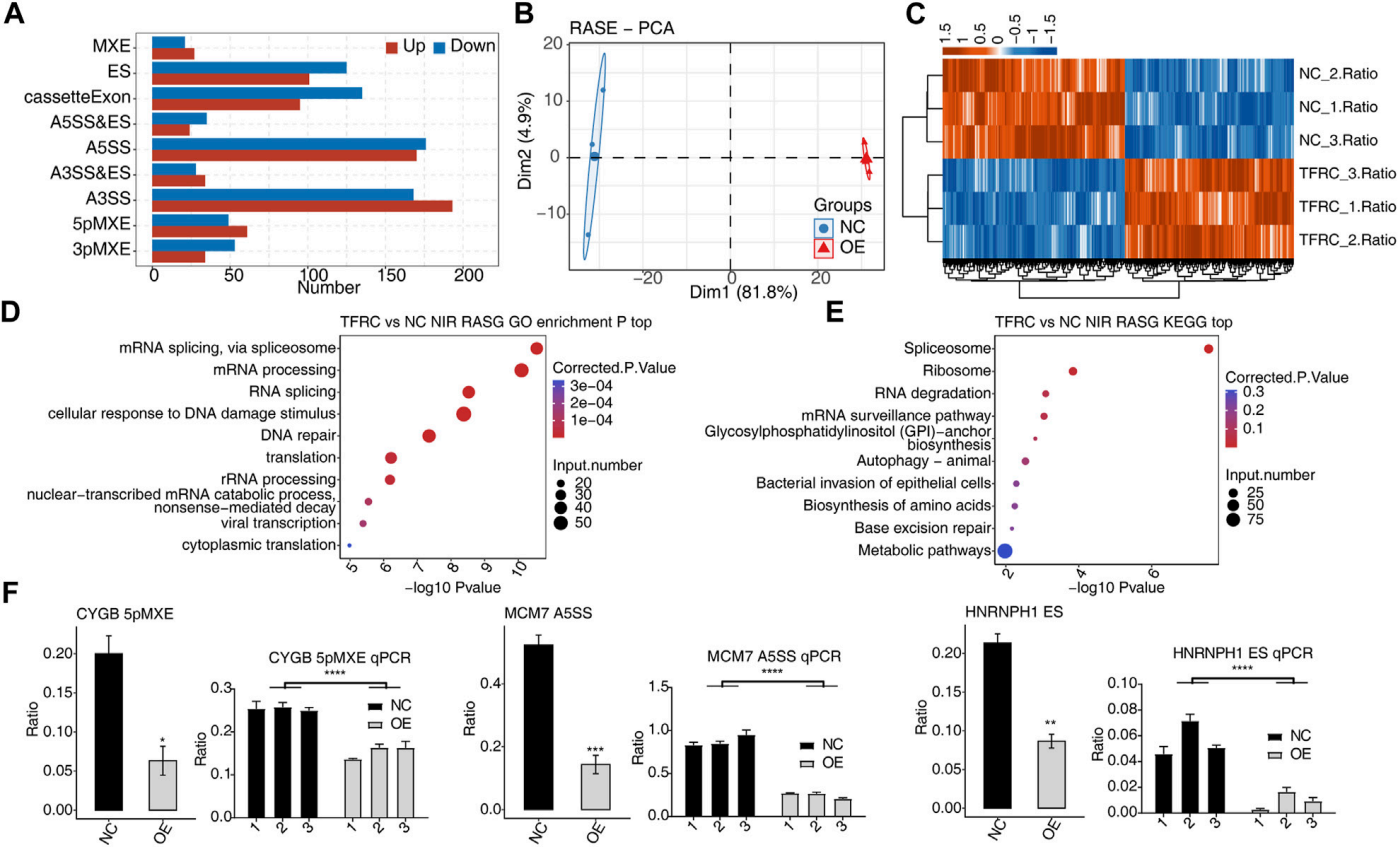
TFRC globally regulates the AS pattern in HRMCs. **(A)** Bar plot showing the number and frequency distribution of regulated ASE types by TFRC-OE in HRMCs. **(B)** PCA results showing the clear separation for the two groups by analyzing the RASE ratios. **(C)** Hierarchical clustering heatmap showing the distribution pattern of all RASE ratios. **(D)** Bubble plot showing the top 10 enriched GO BP pathways for RASGs. **(E)** Bubble plot showing the top 10 enriched KEGG pathways for RASGs. **(F)** Bar plot showing the RNA-seq (left) and RT-qPCR (right) validation results for selected RASEs. Error bars represent SEM. *** *p*-value <0.001, ** *p*-value <0.01, and *p-value <0.05; Student’s *t*-test.

Dysregulated ASEs in TFRC-OE HRMCs were investigated, and 1529 TFRC-regulated ASEs were identified. Four dominant RASE types, including 361 A3SS events, 346 A5SS events, 230 cassette exon events, and 226 ES events, were found (Figure 2A). By analyzing the AS ratios of these RASEs, we found that the first principal component separated TFRC-OE and NC samples, suggesting that they have distinct AS profiles. Heatmap, presenting RASE ratios, showed the consistent ratio changes between TFRC-OE and NC samples with three biological replicates (Figures 2B, 2C). To decipher the underlying mechanisms of these RASEs, we investigated the enriched pathways of genes from RASEs (RASGs) using GO and KEGG databases. Interestingly, the enriched GO BP pathways were RNA splicing-associated pathways, DNA damage and repair pathways, and translation-associated pathways (Figure 2D), and similar results were obtained from KEGG pathway analysis (Figure 2E). Thus, the overexpression of TFRC may modulate spliceosomal and ribosomal RBP splicing activity, indicating a mechanism by which TFRC affects RASEs. Three RASEs were selected, 5pMXE from *CYGB*, A5SS from *MCM7*, and ES from *HNRNPH1*, for RT-qPCR with specific primers complementary to splicing junctions, and all were shown to have altered expression under the OE condition (Figure 2F). Thus, TFRC is observed to have an effect on HRMC AS regulation.

### 3.3 Binding of TFRC to IgAN-associated mRNAs in HRMCs

**FIGURE 3:**
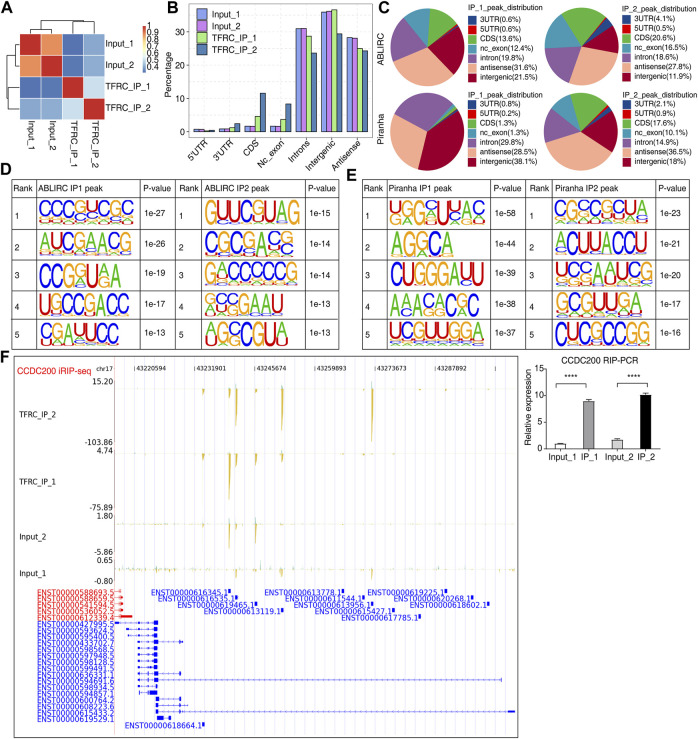
Characterization of the TFRC–RNA interaction profile by iRIP-seq. **(A)** Sample clustering heatmap showing the separation between IP and input samples. **(B)** Bar plot showing the genomic distribution of reads across the reference genome. **(C)** Pie chart showing the genomic distribution across the reference genome of TFRC-bound peaks by two peak calling methods. **(D,E)** Motif presentation by HOMER software showing the top five motifs bound by TFRC. **(F)** IGV-sashimi and bar plots showing the reads distribution of iRIP-seq and RIP-qPCR data for the gene *CCDC200*. Left panel showed the TFRC-binding peak for CCDC200; the right panel showed the RIP-qPCR result. Error bars represent SEM. **** *p*-value <0.0001; Student’s *t*-test.

TFRC modulates gene expression and AS profiles perhaps by binding to the transcripts of DEGs and RASGs, respectively. RIP-seq and high-throughput sequencing methods were employed to identify RBPs’ RNA targets (Wheeler et al., 2018). iRIP-seq was conducted to identify TFRC-bound RNA targets and the protein–RNA interacting sites (Tu et al., 2020). Genome mapping and sample correlation analysis showed the clustering of two IP samples, which were separated from the background input (total RNA without immunoprecipitation) samples (Figure 3A). Peaks found from ABLIRC and analyzed using Piranha software were considered to predict binding sites of TFRC on primary RNAs and occurred in intron, intergenic, and antisense regions. IP samples had higher percentages in non-coding exon (nc_exon) and coding sequence (CDS) regions, and a lower percentage in intron regions (Figures 3B, C). The identification of enriched motifs within the TFRC peaks using HOMER software revealed CG-rich motifs (Figures 3D, E). The analysis of the read distribution of iRIP-seq and RIP-qPCR data for gene *CCDC200* showed several binding sites in one intron region (Figure 3F, left and right panels). Three additional genes, *ANXA2*, *ANKRD30BL*, and *RP11-160E2.6*, were also evaluated, and *TFRC* bound to *ANXA2* exon regions (Supplementary Figures S1A), suggesting binding to mature transcripts. Potential binding sites were also identified in the 5’ exon regions of *ANKRD30BL* (Supplementary Figures S1B) and *RP11-160E2.6* (Supplementary Figures S1C).

### 3.4 TFRC binding to mRNA-regulated AS

**FIGURE 4:**
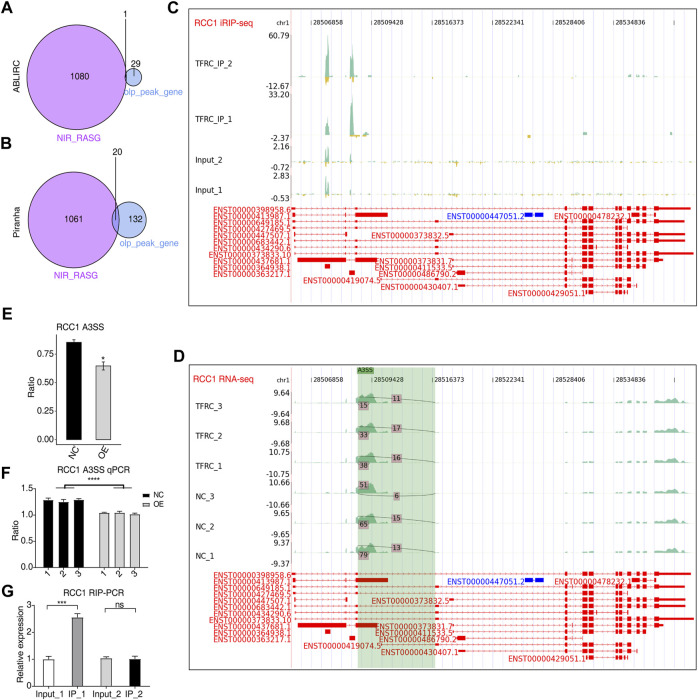
TFRC could regulate AS by binding to primary RNA transcripts. **(A,B)** Venn diagram showing the overlapped genes between RASGs and TFRC-bound peak genes predicted by two methods. **(C,D)** IGV-sashimi plots showing RCC1 peak reads distribution in iRIP-seq **(C)** and AS sites in RNA-seq data **(D)**. **(E)** Bar plot showing alterations to AS ratios and statistical differences of RCC1 A3SS events. **(F)** RT-qPCR result confirming alterations to AS ratios and statistical differences of RCC1 A3SS events. **(G)** Bar plot showing TFRC-binding density to RCC1 transcripts by RIP-qPCR experiments. Error bars represent SEM. * *p*-value <0.05; *** *p*-value <0.001; **** *p*-value <0.0001; Student’s *t*-test.

**FIGURE 5:**
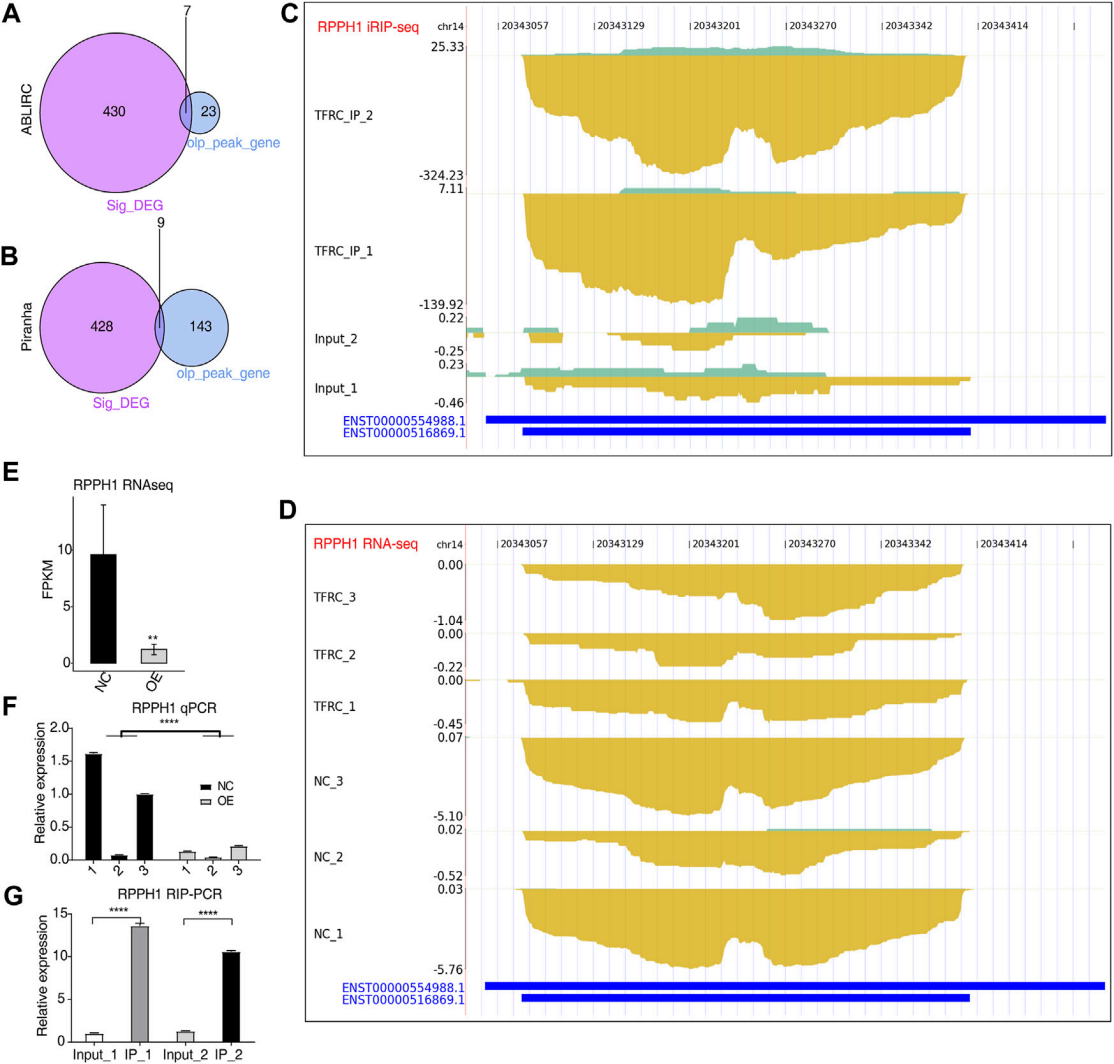
TFRC could bind to primary transcripts to regulate the expression of RNA-encoding genes. **(A,B)** Venn plots showing the overlap of DEGs and TFRC-bound peak genes predicted by two methods. **(C,D)** IGV-sashimi plots showing RPPH1 peak reads distribution from iRIP-seq data **(C)** and expression levels from RNA-seq data **(D)**. **(E)** Bar plot showing the expression pattern and statistical differences of RPPH1 between TFRC-OE and NC samples. **(F)** Bar plot showing the RT-qPCR results and statistical differences of RPPH1 between TFRC-OE and NC samples. **(G)** Bar plot showing the TFRC-binding signal to RPPH1 by the RIP-qPCR experiment. Error bars represent SEM. ** *p*-value <0.01; **** *p*-value <0.0001; Student’s *t*-test.

To further investigate how TFRC modulates the fate of its bound transcripts, we made an overlap analysis between these transcripts and RASGs. In total, 20 overlapping genes were identified using Piranha (Figure 4B) and one from ABLIRC (Figure 4A). Among the overlapping genes, RCC1, involved in the cell cycle and cell physiological activities (Ren et al., 2020), was selected. Furthermore, two TFRC-bound peaks were identified in intronic and exonic regions (Figure 4C). Altered AS ratios were found for *RCC1* in TFRC-OE cells (Figure 4E), and A3SS events with a novel splicing junction with the potential to alter coding sequences are shown in Figure 4D. The sites of AS events were found to be adjacent to TFRC-binding sites, suggesting the regulation of the A3SS events of *RCC1* when TFRC binds to intron and exon regions of primary transcripts (Figure 5C). RT-qPCR experiments in TFRC-OE cells confirmed the regulation of *RCC1* ASEs (Figure 4F) and RIP-qPCR that *RCC1* transcripts were enriched in the IP_1 sample (Figure 4G). Thus, it is likely that TFRC regulated AS patterns by binding to primary transcripts of target genes.

### 3.5 TFRC binding to RNA-regulated expression

To determine whether TFRC regulates gene expression by binding to the targeted RNAs in HRMCs, we overlapped TFRC-regulated DEGs and TFRC-bound RNA genes. Among TFRC-bound RNA genes detected by ABLIRC and Piranha, seven and nine genes were found overlapped with DEGs, respectively (Figures 5A, B). These included *RPPH1*, an RNA component of the RNase P ribonucleoprotein, which has been linked to mesangial cell inflammation and proliferation in diabetic nephropathy (Zhang et al, 2019b). It was tightly bound by TFRC in HRMCs. The density of reads of iRIP-seq was indicated in TFRC IP samples; the binding signals were on *RPPH1*’s short transcripts, while in input samples, very few signals were observed (Figure 5C). The *RPPH1* expression level significantly decreased in TFRC-OE samples in comparison with NC samples (Figure 5D). There were also significant differences in FPKM values between TFRC-OE and NC samples (Figure 5E), and RT-qPCR produced similar results (Figure 5F). Thus, TFRC-OE is observed to inhibit *RPPH1* expression perhaps by binding TFRC to transcripts, promoting their degradation. TFRC-binding signals were also significantly enriched in TFRC IP samples (Figure 5G).

## 4 Discussion

The common primary glomerular disorder, IgAN (Rodrigues et al., 2017), is characterized by glomerular complexes of galactose-deficient IgA1 (Gd-IgA1), and pathogenic molecular mechanisms remained unclear. The IgA1 receptor, TFRC, controls the entry of iron into cells (Gammella et al., 2017) and may have a molecular function in IgAN. Transcriptomic and interactomic analyses of HRMCs overexpressing TFRC revealed that TFRC promoted the expression of cell cycle-associated genes and inhibited expression of cell adhesion-associated genes. TFRC overexpression also led to changes in AS patterns of DNA damage response genes and some gene transcripts, in which bound TFRC were linked to IgAN pathogenesis. The current study indicates that the binding of TFRC to RNA may modulate IgAN progression.

Mesangial deposition, glomerular injury, cellular proliferation, and ECM formation, occur in the late stage of IgAN (Mestecky et al., 2013; Lai et al., 2016; Rodrigues et al., 2017). The observation of upregulated genes associated with the cell cycle and proliferation pathways in TFRC overexpressing cells indicates the stimulation of cell proliferation by TFRC that may be induced by Gd-IgA1 stimulation of cytokines, IL-6, and TGF-β, leading to ECM formation (Tamouza et al., 2007; Lai et al., 2016). *KIFC1*, *CENPH*, *TOP2A*, and *FOXM1* transcripts were also bound by TFRC and have been associated with kidney cell proliferation. The silencing of *KIFC1* inhibited cell proliferation and arrested the cell cycle at the G2/M phase in renal cell carcinoma (RCC) (Li et al., 2018). The active kinetochore component and RCC biomarker, CENPH, inhibited cell proliferation, and promoted cell apoptosis (Wu et al., 2015). In addition, TOP2A is a prognostic RCC marker which promotes clear cell proliferation and migration (Zhang et al, 2019a). The transcription factor (TF), FOXM1, promoted multi-Wnts expression, increasing fibrosis of obstructed kidneys (Xie et al., 2021). Downregulated DEGs included *FABP4*, *ID1*, *KIF20A*, *ATF3*, *H19*, *IRF7*, and *H1-2,* of which *ATF3* and *IRF7* are TFs known to promote the death/apoptosis of renal cells (Jiang et al., 2010) or to inhibit cell proliferation (Lin and Cai, 2020). In summary, the indications are that TFRC may promote the proliferation of mesangial cells via its effects on gene expression and stimulate IgAN progression. Genes shown to be downstream targets of TFRC have not all been previously associated with IgAN but may be involved in Gd-IgA1-induced cell proliferation. Further investigation of the TFRC-regulation of gene expression in HRMCs is required to clarify the situation.

AS is regulated by spliceosome complexes, containing splicing-associated RBPs and RNAs (Wilkinson et al., 2020), and AS profiles were altered in TFRC overexpressing HMRCs. Aberrant AS patterns have been linked to many disease states, including chronic kidney disease (Kim et al., 2018) (Stevens and Oltean, 2016). TFRC has been shown to modulate AS profiles in HeLa cells (Huang et al., 2022), and 1,529 RASEs were identified by TFRC overexpressing cells of the present study, suggesting that the TFRC modulation of AS may be a general phenomenon. TFRC has no canonical RNA-binding domains (RBDs) and is not known to be a spliceosome component, but RASGs were shown to be enriched in mRNA splicing, translation, and DNA damage/repair pathways. A similar regulatory mechanism has been reported for another potential RBP, KRT18, in gastric cancer (Chen et al., 2021). RASEs within the genes, *MCM7*, *CYGB*, and *HNRNPH1,* followed a consistent pattern when analyzed by RT-qPCR. Previous studies have shown that CYGB played an antioxidant and antifibrotic role in kidneys (Nishi et al., 2011), *MCM7* was a proliferative marker for clear cell RCC (Zhang et al., 2021), and HNRNPH1 regulated splicing, expression, MAPK signaling, and ubiquitin-mediated proteolysis (Uren et al., 2016). Thus, there is reason to believe that AS is involved in IgAN pathogenesis and that TFRC may act as an RBP and RASE regulator, although further mechanistic studies are required.

A total of 30 and 152 TFRC-bound genes were identified by ABLIRC and Piranha analysis of iRIP-seq data. TFRC may bind to a small set of transcripts in HRMCs, consistent with its lack of conventional RBDs. RBPs are known to regulate RNA metabolism and functions (Hentze et al., 2018), and the gene *RCC1*, which bound TFRC and was involved in RASGs, was found to influence the G1/S transition of mitotic cell cycle, regulation of mitotic mitosis, and spindle tissue (Hadjebi et al., 2008; Ren et al., 2020). Aberrant AS may change the isoforms and protein structures of the RCC1 product, affecting its function in the cell cycle. The long noncoding RNA (*lncRNA*) gene, *RPPH1*, was also identified as TFRC-binding and among DEGs. *RPPH1* promoted mesangial cell inflammation and proliferation in diabetic nephropathy (Zhang et al, 2019b; Yoo et al., 2019). TFRC may also facilitate IgAN development by binding RPPH1, and influencing inflammation and proliferation of mesangial cells. These results provide further support for the view that TFRC binds RNA targets to modulate AS and facilitate IgAN development.

We acknowledge some limitations to the present study. Polyadenylated RNAs were purified, thus excluding RNAs without polyadenylation. Allowing a maximum of four mismatches obtained a higher mapping ratio but may have reduced the accuracy and reliability of the results by introducing low-quality reads. Larger sample sizes and the validation in clinical samples or animal models are necessary to confirm and enhance the present conclusion. The validation of TFRC-interacted RNAs also requires confirmation by additional experiments. Further experiments are planned to explore the underlying molecular mechanisms of TFRC in IgAN development.

In conclusion, molecular targets and potential functions of the RBP, TFRC, in HRMCs are presented. Targets and pathways were relevant to the molecular mechanisms of IgAN, suggesting that overexpression of the IgA1-TFRC complex altered downstream gene expression and regulated pathogenic processes. TFRC targets show therapeutic potential for IgAN treatment.

## Data Availability

Data available within the article or its supplementary materials. The sequencing data presented in the study are deposited in the GEO database repository, accession number GSE223358.
