# Supplementary material for: TFRC–RNA interactions show the regulation of gene expression and alternative splicing associated with IgAN in human renal tubule mesangial cells
Source: Front Genet. 2023 Jul 20;14:1176118. doi: 10.3389/fgene.2023.1176118 (PMC10397801; doi:10.3389/fgene.2023.1176118)
Supplement: Supplementary file 1 [file Table1.pdf]

Supplementary Material

• TFRC-RNA interactions show regulation of gene expression and alternative splicing associated with IgAN in human renal tubule mesangial cells

Jian-Si Li[1*], Xiao Chen[2], Ailing Luo[3] and Dong Chen[3]

*** Correspondence:** Jian-Si Li: lijiansimm@126.com

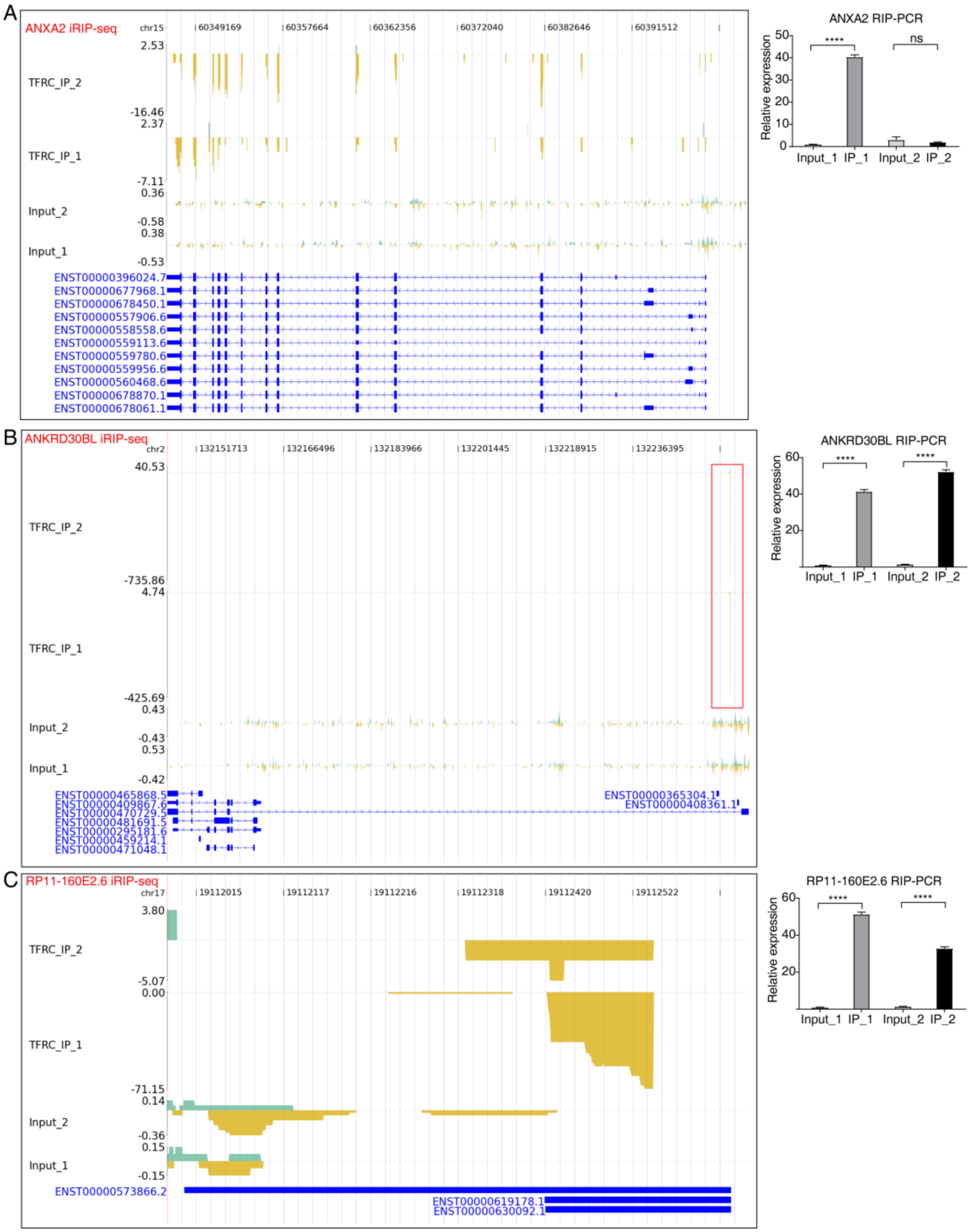

Supplementary Figure 1. (A-C) TFRC binding peak of *ANXA2* (A), *ANKRD30BL* (B) and *RP11-160E2.6* genes (C). IGV-sashimi plot showing *ANXA2*, *ANKRD30BL* and *RP11-160E2.6* (left). Quantification of *ANXA2*, *ANKRD30BL* and *RP11-160E2.6* expression by qRT-PCR using iRIP-seq data (right). Reads distribution is plotted in the upper panel and gene transcripts in the lower.

Table S1: qRT-PCR primers for gene expression qualification.

Gene	Primer	Sequence (5'-3')
GAPDH	Forward	GGTCGGAGTCAACGGATTTG
	Reverse	GGAAGATGGTGATGGGATTTC
TFRC	Forward	ATTGAACCTGGACTATGAGAG
	Reverse	TGGAAGTAGCACGGAAGA
